# Nutrition in Cancer Patients

**DOI:** 10.3390/jcm8081211

**Published:** 2019-08-14

**Authors:** Paula Ravasco

**Affiliations:** 1 University Hospital of Santa Maria, 1649-035 Lisbon, Portugal; p.ravasco@medicina.ulisboa.pt; 2 University of Lisbon, 1649-028 Lisbon, Portugal; 3 Centre for Interdisciplinary Research in Health (CIIS) of the Portuguese Catholic University, 1649-023 Lisbon, Portugal

**Keywords:** cancer, nutrition, nutritional therapy, nutritional support, malnutrition, cachexia, sarcopenia, survival

## Abstract

**Background:** Despite being recognised that nutritional intervention is essential, nutritional support is not widely accessible to all patients. Given the incidence of nutritional risk and nutrition wasting, and because cachexia management remains a challenge in clinical practice, a multidisciplinary approach with targeted nutrition is vital to improve the quality of care in oncology. **Methods:** A literature search in PubMed and Cochrane Library was performed from inception until 26 March. The search consisted of terms on: cancer, nutrition, nutritional therapy, malnutrition, cachexia, sarcopenia, survival, nutrients and guidelines. Key words were linked using “OR” as a Boolean function and the results of the four components were combined by utilizing the “AND” Boolean function. Guidelines, clinical trials and observational studies written in English, were selected. Seminal papers were referenced in this article as appropriate. Relevant articles are discussed in this article. **Results:** Recent literature supports integration of nutrition screening/assessment in cancer care. Body composition assessment is suggested to be determinant for interventions, treatments and outcomes. Nutritional intervention is mandatory as adjuvant to any treatment, as it improves nutrition parameters, body composition, symptoms, quality of life and ultimately survival. Nutrition counselling is the first choice, with/without oral nutritional supplements (ONS). Criteria for escalating nutrition measures include: (1) 50% of intake vs. requirements for more than 1–2 weeks; (2) if it is anticipated that undernourished patients will not eat and/or absorb nutrients for a long period; (3) if the tumour itself impairs oral intake. N-3 fatty acids are promising nutrients, yet clinically they lack trials with homogeneous populations to clarify the identified clinical benefits. Insufficient protein intake is a key feature in cancer; recent guidelines suggest a higher range of protein because of the likely beneficial effects for treatment tolerance and efficacy. Amino acids for counteracting muscle wasting need further research. Vitamins/minerals are recommended in doses close to the recommended dietary allowances and avoid higher doses. Vitamin D deficiency might be relevant in cancer and has been suggested to be needed to optimise protein supplements effectiveness. **Conclusions:** A proactive assessment of the clinical alterations that occur in cancer is essential for selecting the adequate nutritional intervention with the best possible impact on nutritional status, body composition, treatment efficacy and ultimately reducing complications and improving survival and quality of life.

## 1. Introduction

Cancer is a complex disease that results from multiple interactions between genes and the environment, and is regarded as one of the current leading causes of mortality worldwide [1,2]. Metabolic and nutritional alterations can influence survival and recovery of cancer patients: malnutrition, sarcopenia and cachexia [3,4]. Malnutrition ensues from an inflammatory state that promotes anorexia and consequently, weight loss. It is highly prevalent in cancer patients [5] as 15 to 40% of patients report weight loss at diagnosis [6]. It is estimated that 40 to 80% of all cancer patients will be malnourished during the course of the disease. Furthermore, malnutrition can influence treatment outcomes, delay wound healing, worsen muscle function and increase the risk of post-operative complications. It can also impair tolerance and response to antineoplastic treatments, which can in turn lead to extended hospital stay, increase the risk for treatment interruptions, and possible reduced survival [7,8]. Sarcopenia is characterised by a decrease in lean body mass with an impact both on strength and physical function that may decrease the quality of life [9]. As cancer-related weight loss in obese patients cannot be identified by a low body mass index (BMI), sarcopenic obesity, defined as low lean body mass in obese patients, is frequently overlooked [10]. In these patients, changes in body composition result in an increased metabolic risk, and it seems to be a significant predictor of treatment related adverse events [11,12]. Cancer cachexia is a complex multifactorial syndrome that results from a combination of metabolic alterations, systemic inflammation and decreased appetite. It is characterised by an involuntary sustained weight loss and loss of skeletal muscle mass, with or without loss of fat mass that are irreversible by conventional nutritional support [13].

In addition to the disease, antineoplastic treatments and/or surgery have a significant impact on patients’ nutritional status [14,15,16]. During chemotherapy (CT), more than 50% of patients experience dysgeusia, nausea, vomiting and mucositis, and radiotherapy (RT) related complications are also common. It is also established that poor nutritional status increases surgical morbidity and post-surgical complications [17]. Nutritional intervention in cancer patients aim to identify, prevent and treat malnutrition through nutritional counselling with or without oral nutritional supplements (ONS) or via artificial nutrition, i.e., enteral or parenteral nutrition [18,19,20], as well as to address metabolic and nutritional alterations that influence patients’ recovery and survival [19,20]. Despite the fact that nutritional intervention is a key component, nutritional support is not widely accessible to all patients at nutritional risk [21,22,23]. Additionally, given the incidence of nutritional risk in cancer and the fact that the management of cachexia remains a challenge in clinical practice [24], a multidisciplinary approach is vital to define efficient strategies that can improve quality of care in cancer patients. According to the reviewed data and guidelines, nutritional intervention should be central and adjuvant to any treatment and should be included in the multidisciplinary approach mandatory in oncology. This will allow for more adequate and efficient results in these patients. Multidisciplinary follow-up, with early and regular nutritional intervention, is of major importance in oncology, thus being a key factor for successful treatment and recovery. The present article aims to provide insights and an overview of the most recent literature regarding key nutritional aspects in cancer patients.

Based on this framework, a literature search in PubMed and Cochrane Library was performed from inception until 26 March. The search consisted of terms: cancer, nutrition, nutritional therapy, malnutrition, cachexia, sarcopenia, survival, nutrients, guidelines. Key words were linked using “OR” as a Boolean function and the results of the four components were combined by utilizing the “AND” Boolean function. Guidelines, clinical trials and observational studies written in English, were selected. Seminal papers in the area, even if dated outside the search timeline, were referenced in this article as appropriate. 

## 2. Results

### 2.1. Nutritional Screening and Assessment

Screening for nutritional risk as early as possible allows for the identification of patients at risk of becoming malnourished [25]. Screening should be done as early as possible, and recent literature suggests that it should be done at diagnosis or at hospital admission; screening should be repeated in the course of treatment for referral for evaluation if needed [19,21,23,25,26,27]. Evidence supports the integration of malnutrition screening in cancer patients care. The adequate tool for screening undernutrition should be brief and easy to fill, inexpensive, highly sensitive and have good specificity [25]. MUST (Malnutrition Universal Screening Tool) and NRS-2002 (Nutritional Risk Screening-2002) are considered suitable [28,29,30]; the MNA (Mini Nutritional Assessment) is a suitable tool for nutritional assessment in the senior population [19,23]. 

When nutritional risk is present, screening should be followed by comprehensive nutritional assessment to better determine the course of nutritional intervention. It seems there is no consensus on the best method to perform this assessment, but SGA (Subjective Global Assessment) and PG-SGA (Patient Generated-Subjective Global Assessment) have been validated for nutritional assessment of adult oncology patients [25,26,31]. 

When used isolated, weight loss is ineffective to detect malnutrition, as it has low sensitivity for metabolic changes that occur in cancer patients. Yet, its early and regular assessment, combined with the evaluation of nutritional intake, BMI and inflammatory status is a standard clinical recommendation [19,26]. As for BMI, it has low sensitivity to detect changes in the nutritional status, especially in obese patients, thus it should only be used combined with other assessment tools [26,32]. 

Body composition provides valuable information in the management of cancer patients, as imaging methods detect loss of muscle mass as well as fatty muscle infiltration [2]. In cancer patients at risk for malnutrition, sarcopenia and cachexia, muscle mass should be assessed [19,21]. Methods available are dual X-ray absorptiometry (DEXA), computed tomography scans at the level of the 3rd vertebra or bioimpedance analysis (BIA). Additionally, it has been recommended that nutritional assessment should be performed for the stages of cancer cachexia, as nutritional intervention is most effective in the stages of precachexia and cachexia [13]. 

### 2.2. Nutritional Intervention

In order to tackle nutritional deterioration, gathering objective data on nutritional status and its evolution throughout the disease course is of prime concern. Different cancer types or locations display different nutritional patterns that require tailored nutritional therapy. Nutritional deterioration is a multifactorial end-result determined by cancer-related and nutrition- and/or metabolic-related factors. Proper nutrition can alleviate symptom burden, improve health across the cancer continuum, support cancer survivorship [33,34,35,36] and is a hallmark of successful cancer treatment. 

Nutritional interventions will vary according to patients’ medical history, type and stage of cancer, as well as to the response to treatment. If the patient can eat and has a functional gastrointestinal tract, nutritional counselling, with or without ONS should be the elected intervention to address altered nutritional demands due to treatment or disease [19,21,26]. ONS may be necessary, as a means to compensate for lower food intake and to try to prevent nutritional deterioration during the course of treatments. Monitoring compliance with the selected nutritional intervention is essential.

#### 2.2.1. Individualised Nutritional Counselling

**Figure 1:**
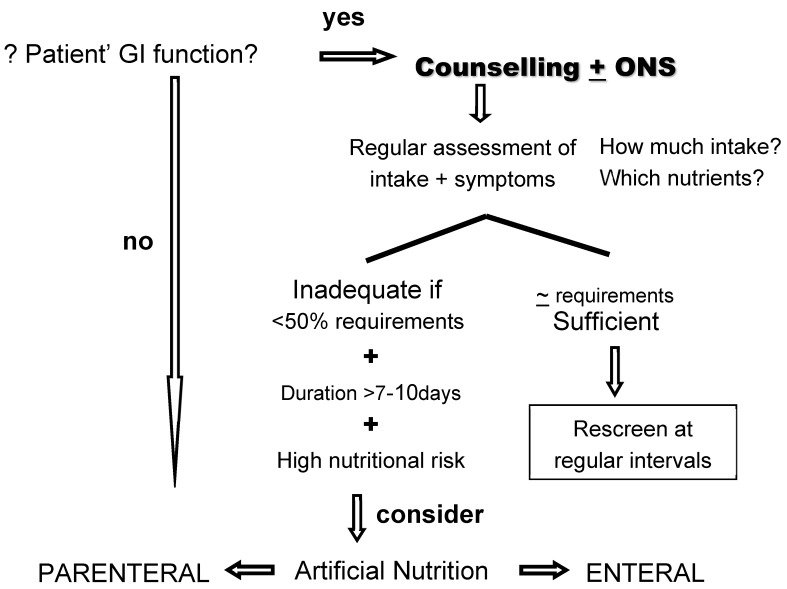
Evidence based decision making plan.

In clinical practice, oral nutrition is always the priority. Oral nutrition is the preferred route of feeding as it is a significant part of the patient’s daily routine and does contribute substantially to the patients’ autonomy [19]. It represents a privileged time to spend with family and friends, avoiding the tendency for isolation. The acknowledgement that the prescribed diet is individualized, adapted and adequate to individual needs, empowers the patient with a feeling of control, thus it is also a highly effective approach for psychological modulation. All these factors may potentially contribute to improve the patients’ quality of life, and may modulate acute and late treatment morbidity. The referral for a nutrition professional responsible for the individualised dietary counselling should always be based on decision-making plans (Figure 1). 

As clinicians we have to recognise the dimensions that are determinant for the patients. Indeed, the diet is the only factor that the patient feels he/she can control during the whole course of treatments and interventions. Also, an adequate food intake is recognised by the patient as well as by the family and caregivers, as essential to maintain the daily activity, energy, functional capacity and to overcome treatments more successfully. Notably, nutritional wasting is common regardless of the cancer stage (curative, adjuvant, to palliative) and is an independent predictor of poor physical function, lower quality of life, surgical complications, and reduced survival [3,10,13,37]. Cancer wasting is characterised by muscle mass deterioration that occurs in more than 50% of newly diagnosed cancer patients, in comparison with 15% prevalence in healthy individuals of similar age [38]. Since both muscle mass and adipose tissue play a role in oncological outcomes, strategies to optimize body composition are an important part of successful cancer therapy. Hence, a major goal of nutrition intervention is to favourably influence body composition, with the potential to improve cancer therapy outcomes, morbidities and ultimately, prognosis.

To be effective, individualised counselling has to be based on a thorough assessment of various nutritional and clinical parameters: nutritional status and dietary intake, usual dietary pattern, intolerances or food aversions, patients’ psychological status, autonomy, cooperation, need for help or support of others in the act of eating. A thorough symptom assessment is also mandatory (Table 1).

Individualised nutritional counselling taking into consideration patients’ clinical condition and symptoms, was the most effective nutrition intervention, assuring a sustained and adequate diet, which was able to overcome the predictable deterioration subsequent to RT [35,36,39,40,41,42]. Positive effects were experienced in the long term with a possible impact of patients’ prognosis [43] as recently showed in a randomized trial, the preliminary results of which were presented in the ESPEN Congress in 2018. Another randomised trial of nutritional therapy showed that intervention had an impact in maintaining patients’ nutritional status and function [39]. In this study, individualised intensive nutrition counselling was compared with individualised on-demand nutrition counselling by a dietician prior to and during oncologic treatment. On-demand nutrition counselling requested by physician/nurse referral, seemed not inferior to intensive counselling; thus, these results do emphasise the importance of establishing multimodal nutrition teams to effectively and timely screen and orient patients for adequate nutrition [39]. Several guidelines to date do include nutritional counselling as their standard of care for malnourished patients or at risk of malnutrition [19,20,21,23,26,27] or during anti-neoplastic treatments in head-neck (HNC), oesophageal and colorectal cancers as these patients are in particular risk of malnutrition due to tumour location and irradiated area [14]. 

If/when oral nutrition is inadequate/insufficient, artificial nutrition should be considered [19,20,21,23,26,27]. Criteria for the escalation in nutritional measures are: (1) inadequate food intake (<50% of requirements) is anticipated for more than 10 days due to surgery or chemotherapy (CT)/radiotherapy (RT); (2) if food intake is less than 50% of the requirements for more than one to two weeks; (3) if it is anticipated that undernourished patients will not be able to eat and/or absorb the adequate amount of nutrients for a long period time, due to antineoplastic treatments; (4) if the tumour mass itself impairs oral intake and food progression through the upper GI tract. The decision between enteral nutrition (EN) and parenteral nutrition (PN) must take into account the site of the tumour, its extent, complications, treatment plan and intent, prognosis, patients’ overall physical status and the duration of the nutritional support [19,20,21,23,26,27,44].

#### 2.2.2. Artificial Nutrition

If the intestinal functions are preserved, EN should be preferred in order to maintain gut integrity and reduce bacterial translocation [45], as well as to reduce infectious complications [19,20,21,23,26]. A standard polymeric feeding formula should be preferred. EN is recommended in undernourished or at-risk patients during CT if undernutrition is present or if inadequate food intake is present or anticipated [19,22,23,27,46]. Systematic artificial nutrition during CT treatment is not recommended [19,20,21,23,26]. In radiation-induced severe mucositis or in obstructive tumours of the head-neck or thorax, either PEG or nasogastric tube are recommended [19,20]. EN is contraindicated in: intestinal obstruction or ileus, severe shock, intestinal ischaemia, high output fistula, severe intestinal haemorrhage, intestinal insufficiency due to radiation enteritis, short bowel syndrome, peritoneal carcinomatosis chylothorax [19,21,23,26]. In these situations, or whenever EN is insufficient, a combination of EN and PN or PN alone should be considered [19,21,23,26]. As for PN, it should be initiated early [19,21,23,26] whenever indicated. PN is the first option of nutritional support in cases of intestinal failure; whenever macro and micronutrient’ requirements can only be fulfilled via the parenteral route, long term artificial nutrition as home parenteral nutrition (HPN) is standard recommendation [19,44,47,48]. 

As for the macronutrients in PN, amino acids (AA) requirement of cancer patients relies on: negative balance between whole body protein synthesis and breakdown, doses of AA closer to 2 g/kg/day may be required to control catabolism and stimulate synthesis vs. 0.8 g/kg/day as recommended for healthy subjects [49], and for older subjects and chronic disease, most recent clinical guidelines recommend >1.0 g/kg/day of protein. Hence, to support protein balance, up to 1.5 g/kg/day or more of protein is the consensual recommendation. In the nutritive PN admixtures, essential AA should be present in approximately 50% of AA and branched chain AA should account for the remainder 50% of total AA [50]. In what concerns fat as an energy substrate, the most consensual regimens have fat accounting for ≈50% of non-protein calories [51,52].

Recently, PN as a supplemental route of nutrient administration (SHPN) emerged as a possible resource to optimise nutrient delivery. Prospective studies [53,54,55,56,57] on SHPN suggest a possible benefit in energy balance, increased body fat, greater maximum exercise capacity and QoL. A recent randomised trial showed that SHPN may prevent loss of MM in patients with incurable gastrointestinal cancer [57]. Hence, there is yet insufficient evidence to recommend SHPN in cancer patients to improve QoL and nutrition parameters. Additionally, practice of HPN differs between countries; most do not consider the use of PN if there is a functional gastrointestinal tract, while others may consider its use if it is according to the will of the patient [58,59,60]. 

Refeeding syndrome can occur when severe shifts in fluids and electrolytes happen in severely malnourished patients receiving EN or PN, and it may cause hypophosphatemia, hypokalaemia, hypomagnesaemia, thiamine deficiency, changes in sodium, glucose and fluid balance and also in protein and lipid metabolism [19,21]. Its prevention is recommended when BMI < 16 kg/m^2^ or in the presence of unintentional weight loss >15% within the last three to six months or whenever there is little or no nutritional intake for more than 10 days or if there are decreased levels of potassium, phosphate or magnesium prior to feeding. If a severe decrease in food intake occurs for at least five days, it is recommended a gradual increase in nutrition over several days, and no more than 50% of the calculated energy requirements should be supplied during the first two days of feeding [19,25]. The identified fluid and electrolytes imbalances should be corrected, and the circulatory volume, fluid balance, heart rate and rhythm, as well as clinical status, should be monitored closely. Attention to the refeeding syndrome risk is currently contemplated in guidelines for cancer management [19,20,21,22,23,26,27].

#### 2.2.3. Surgery

In order to minimise the metabolic stress response and catabolism associated with surgery in undernourished patients, the enhanced recovery after surgery program (ERAS) is recommended for all cancer patients undergoing curative or palliative surgery [18,22,61]. Within ERAS protocol the following principles should be followed: Screening for malnutrition and give additional nutritional support if necessary [18,22]; avoid preoperative fasting; preoperative carbohydrate treatment should be considered as well as the reestablishment of oral feeding on the first postoperative day; and early mobilisation [18,22]. To avoid preoperative fasting, patients with no risk of aspiration, are allowed to eat solid food until six hours and drink clear fluids until two hours before anaesthesia [18]. 

In oncologic surgical patients, with moderate to severe nutritional risk, nutritional support is recommended before and after surgery [18,25]. If severe malnutrition is present, delaying surgery may be necessary [18,25]. When submitted to major surgery, nutritional support should be provided routinely, with particular attention to elderly sarcopenic patients.

Besides the ERAS protocol, an early start of nutritional supplementation can significantly diminish the degree of weight loss and incidence of complications [22,25]. If it is anticipated that after surgery, the patient will be unable to eat for more than seven days, it is advised to start nutrition therapy even in well-nourished patients [18,22,25]. After surgery, oral nutrition should also be preferred to EN and the latter should be preferred to PN. If oral intake is possible, it should start after surgery without interruption, after assessing individual tolerance. If oral nutrition is not possible, EN should be initiated within 24 h, preferring standard polymeric enteral formulae if adequate [25].

#### 2.2.4. Radiotherapy and Chemotherapy

Oral mucositis, dysphagia and diarrhoea are common complications of RT and/or CT treatments [12,13,14,15,16,17,18,19,20,21,22]. During RT, nutritional counselling is also recommended, especially in HNC, thorax and gastrointestinal (GI) tract cancers [12,13,14,15,16,17,18,19,20,21,22,23]. When deemed necessary, ONS should be provided [62], and when severe mucositis is present, artificial nutrition should be considered [23]. When dietary counselling and ONS are insufficient to reduce weight loss or if in the presence of severe mucositis or obstructive tumours of the head or neck or thorax, artificial nutrition should be considered [19,20,23]. In patients treated with RT or chemoradiotherapy, PN is not recommended [19], and it should only be considered when adequate nutrition cannot be assured with oral or EN [19].

### 2.3. Specific Nutrients 

Nutritional strategies that potentially allow better management of cancer have been widely investigated, but few have reached conclusive results. 

#### 2.3.1. Protein

Many patients with cancer do not meet the recommended intake (1.2–1.5 g/kg/day), and not even the one for healthy individuals (0.8 g/kg/day) [63]. Limited protein intake ensues mainly from nutrition impact symptoms that affect dietary intake [64]. Recent guidelines do suggest a higher range of protein intake (1.2–1.5 g/kg/day), because of the positive results of higher protein intake in protein balancing and in maintaining muscle mass. Of additional interest is a recent study showing an inverse association between red meat consumption and seven-year mortality among 992 individuals with stage III colon cancer [65], suggesting that higher protein intake may actually be beneficial in cancer. 

Interventions with amino acids have been tested in cancer, aiming to optimise nutritional status and counteract muscle mass wasting. They include supplementation with branched chain amino acids (leucine, isoleucine and valine) [63], β-hydroxy β-methyl butyrate, carnitine and creatine. Yet further research is needed to clarify potential benefits. 

As for glutamine, its supplementation in cases of oral mucositis or to prevent/treat diarrhoea during pelvic RT, is not recommended [19,22,26]. As for its use when PN is required for patients undergoing haematopoietic stem cell transplant, guidelines are not identical: there is a fair graded recommendation for eventual use of 0.2–0.5 g/kg/day [26], and the indication that there is not enough evidence to recommend for or against glutamine to reduce anticancer therapy side effects, especially in high dose protocols [19]. In what concerns its potential to improve muscle mass, there is not enough data to support it. 

#### 2.3.2. Eicosapentaenoic Acid and Fish Oil 

Eicosapentaenoic acid (EPA) has been identified as a promising nutrient with appointed clinical benefits. Several mechanisms have been proposed to explain the potential benefits of EPA on the body composition: inhibition of catabolic stimuli by modulating the production of pro-inflammatory cytokines and enhancing insulin sensitivity that induces protein synthesis. Intervention studies showed that EPA may attenuate deterioration of nutritional status and may aid in improving calorie and protein intake. Recent systematic reviews found that EPA can reduce inflammation and has a potential to modulate the nutritional status/body composition [66,67]. Furthermore, some studies suggest that n−3 fatty acids inhibit proliferation of cancer cells [68] and might decrease CT toxicity [69]. Given the large number of studies reporting a positive impact of n−3 fatty acids on the muscle mass, it is likely that this would be a practical and effective intervention for preventing loss of muscle without significant side effects [19]. It is noteworthy that the strength of recommendation somewhat differs for the use of n−3 fatty acids supplementation in weight losing cancer patients not responding to standard nutritional therapy. This recommendation has been rated as strong [26] and weak [19]. Nevertheless, both guidelines are inclined to consider supplementation with long chain fatty acids and fish oil to decrease systemic inflammation and improve appetite, food intake and body weight. 

Trials with homogeneous patient populations regarding cancer type, stage, anti-neoplastic regimens, supplement dosage and modality of administration are needed to clarify clinical benefits. Indeed, it is noteworthy that in view of the modest survival benefits of CT/RT in some cancers, important issues for physicians are to optimize well-being, quality of life via nutritional status and adequate body composition [70].

#### 2.3.3. Micronutrients

Because of the adverse effects of therapy and restricted diet of many patients, the American Institute for Cancer Research [71], American Cancer Society [72] and the European Society for Clinical Nutrition and Metabolism—ESPEN [19] support the use of a multivitamin-multimineral supplement in doses close to the recommended dietary allowance. High doses of vitamins and minerals are discouraged in the absence of specific deficiencies [19,20]. Vitamin D deficiency might be relevant in cancer [19]; also, an association has been reported between low vitamin D and muscle wasting. As a consequence, vitamin D may be needed to optimise protein supplements effectiveness. In light of the recent literature, vitamin D supplementation with 600–800 international units (RDA) in cancer patients can be beneficial in the context of preventing muscle wasting, but further research is needed.

## 3. Discussion

In cancer, deterioration and muscle wasting result from the combination of reduced nutrient absorption, alterations in appetite, taste and/or dietary intake, hormone-induced metabolic changes and cancer-related immune activation with cytokine release. Regardless of the underlying mechanisms, cancer-related weight loss is a multidimensional manifestation that worsens patients’ well-being, tolerance to antineoplastic therapies and prognosis. Clinically speaking, weight loss is frequent in cancer patients, and depending on the location of the tumour, it is present in 15 to 40% of cancer patients at diagnosis. Weight loss is frequently the first sign of the nutritional alterations that occur in the course of the disease and is associated with poor prognosis, reduced quality of life and morbidity [62]. Cancer cachexia can be defined as ‘*a multi-factorial syndrome defined by an ongoing loss of skeletal muscle mass (with or without loss of fat mass) that cannot be fully reversed by conventional nutritional support. It leads to progressive functional impairment. Its pathophysiology is characterized by a negative protein and energy balance driven by a variable combination of reduced food intake and abnormal metabolism*’. The agreed diagnostic criterion for cachexia was weight loss >5%, or weight loss >2% in individuals already showing depletion of body weight (BMI < 20 kg/m^2^) or of skeletal muscle (sarcopenia). Assessment for classification and clinical management should also include the following domains: ‘anorexia/reduced food intake, catabolic drive, muscle mass and strength, functional and psychosocial impairment’ [13].

The main nutritional problem in cancer is wasting of muscle mass, acknowledged to be a predictor of lower quality of life, impaired functionality, surgical complications and shortened survival [10,63,73,74]. Of note that sarcopenia occurs independently of loss of weight or of fat mass. Thus, a clinically relevant phenotype that also emerged in cancer is characterised by sarcopenia with excessive fat mass. Additionally, to the previous studies demonstrating the major impact of muscle mass depletion on survival and treatment toxicity [73,74], a recent study in a cohort of head-neck cancer patients, showed that patients with cachexia had a worse disease-free survival compared with non-cachectic patients [12,39]. 

Bearing this in mind, the clinical efforts and priority given to improve treatment outcomes, will logically have to include nutritional intervention and adequacy of body composition. The search for an effective nutritional intervention that improves body composition (preservation of muscle mass and muscle quality) is of utmost importance for clinicians and patients, given the implications for prognosis. Early detection of malnutrition and cachexia should be part of a multimodal approach to improve both patient-centred and oncology outcomes [47]. 

## 4. Conclusions

In the present article, the most recent guidelines for the management of cancer patients, as well as original studies in nutrition and cancer, were included. Nutrition is a central factor in oncology, influencing the development of the disease, tumour inherent symptoms, response to, and recovery after anti-neoplastic treatment(s), thus having a strong impact on the quality of life and prognosis of the disease. A main nutritional feature is wasting of muscle mass, strongly associated with decreased functional capacity, higher incidence of chemotherapy toxicity, increased hospitalization and complication rate, as well as mortality. Nutritional risk screening and assessment in cancer patients allows for the early detection of malnourished patients and also for a prompt nutritional intervention aiming to prevent nutritional deterioration and muscle wasting. A proactive assessment of the clinical alterations that occur during treatments and during the disease course, is essential for selecting the adequate nutritional intervention, aiming for the best impact on patients’ outcomes. Early tailored intervention has the potential to improve body composition and treatment’ efficacy, and as evidence stands, it is an obligatory adjuvant intervention, with the likelihood of improving prognosis of the disease itself.

## Figures and Tables

**Table 1:** Common causes for a poor nutrient intake in cancer patients.

Deterioration in taste, smell and appetite, as a consequence of the tumour and/or therapy Altered food preferences/food avoidance/food aversion Eating problems (teeth, chewing) Dysphagia, odynophagia or partial/total gastrointestinal obstruction Early satiety, nausea and vomiting Soreness, xerostomia, sticky saliva, painful throat, trismus Oral lesions and oesophagitis Radiotherapy/chemotherapy induced mucositis Acute or chronic radiation enteritis during and after radiotherapy Depression, anxiety Pain
